# NMR measurement of biomolecular translational and rotational motion for evaluating changes of protein oligomeric state in solution

**DOI:** 10.1007/s00249-022-01598-w

**Published:** 2022-04-05

**Authors:** Shenggen Yao, David W. Keizer, Jeffrey J. Babon, Frances Separovic

**Affiliations:** 1 Bio21 Molecular Science and Biotechnology Institute, The University of Melbourne, Melbourne, VIC 3010 Australia; 2 The Walter and Eliza Hall Institute of Medical Research, Parkville, VIC 3052 Australia; 3 Department of Medical Biology, The University of Melbourne, Melbourne, VIC 3010 Australia; 4 School of Chemistry, The University of Melbourne, Melbourne, VIC 3010 Australia

**Keywords:** NMR, NMR relaxation, PGSE NMR, Protein oligomeric state, Rotational reorientation, Translational diffusion

## Abstract

Defining protein oligomeric state and/or its changes in solution is of significant interest for many biophysical studies carried out in vitro, especially when the nature of the oligomeric state is crucial in the subsequent interpretation of experimental results and their biological relevance. Nuclear magnetic resonance (NMR) is a well-established methodology for the characterization of protein structure, dynamics, and interactions at the atomic level. As a spectroscopic method, NMR also provides a compelling means for probing both molecular translational and rotational motion, two predominant measures of effective molecular size in solution, under identical conditions as employed for structural, dynamic and interaction studies. Protein translational diffusion is readily measurable by pulse gradient spin echo (PGSE) NMR, whereas its rotational correlation time, or rotational diffusion tensor when its 3D structure is known, can also be quantified from NMR relaxation parameters, such as ^15^N relaxation parameters of backbone amides which are frequently employed for probing residue-specific protein backbone dynamics. In this article, we present an introductory overview to the NMR measurement of bimolecular translational and rotational motion for assessing changes of protein oligomeric state in aqueous solution, via translational diffusion coefficients measured by PGSE NMR and rotational correlation times derived from composite ^15^N relaxation parameters of backbone amides, without need for the protein structure being available.

**Graphical abstract:**

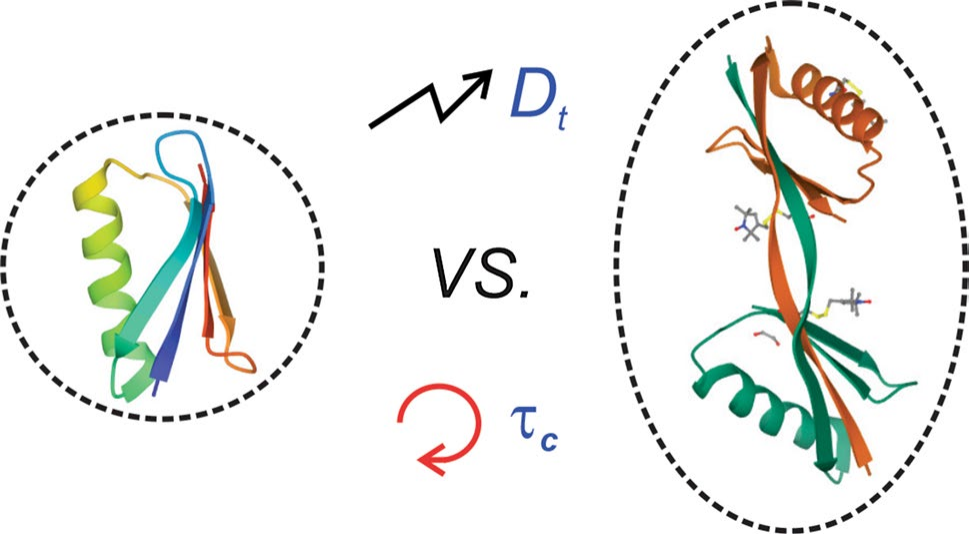

## Introduction

Protein oligomerization is a common event in cellular process where a significant proportion of proteins require oligomerization prior to interacting with their biological partners (Ali and Imperiali 2005). Oligomerization as a prerequisite for function in cells has been reported for many proteins involved in biological processes, e.g. membrane transport proteins and amyloid proteins, where these proteins undergo self-association to form homo-, hetero- or domain-swapped dimers and subsequent functional higher order oligomeric forms. Knowledge of protein oligomeric state in biophysical characterization in vitro is not only significant for the optimization of experimental conditions, but also critical for determining biological relevance of results in vivo. A variety of biophysical methods, with their own inherent advantages and limitations, are available for the characterization of protein oligomeric state. These include size exclusion chromatography, analytical ultracentrifugation, dynamic light scattering, mass spectrometry, and fluorescence microscopy, and their application to studies of protein oligomerization has recently been reviewed (Gell et al. 2012).

Nuclear magnetic resonance (NMR) is well established as a key complementary methodology to X-ray crystallography and cryo-electron microscopy for 3D structure elucidation of proteins and protein complexes, particularly when the systems of interest do not crystalize or are too small for electron microscopy (Sugiki et al. 2017). In addition, NMR is a principal experimental technology for investigating protein dynamics at atomic level across a broad range of time scales (Palmer 2004, 2016). Like other molecules in solution, proteins undergo persistent global motions, namely translation and rotation, as well as segmental and residue-specific motions, including fast atomic fluctuation, sidechain rotation, and large-scale local structural change. Insights into residue-specific protein dynamics gained via NMR spectroscopy have unveiled critical correlations between protein dynamics and their functions (Alderson and Kay 2021). In solution NMR, a first sign of a change in protein oligomeric state may be reflected in the spectral linewidth as a consequence of an effect on overall rotational reorientation. Analyses of NMR spectral linewidth are still employed for evaluation of protein oligomeric state (Bjorndahl et al. 2011). In fact, as a spectroscopic method, NMR readily provides a means for quantifying both translational and rotational motions of proteins, two key size dependent properties of molecules in solution. A previous report correlates molecular mass with rotational correlation time derived from ^15^N relaxation parameters of proteins from a range of studies (Maciejewski et al. 2000). Here, we limit our focus on the fundamentals of NMR measurement of protein translational diffusion coefficients and collective ^15^N relaxation parameters of backbone amides, without the need of knowing its 3D structure, to evaluate change in the protein oligomeric state.

## Background

### Molecular translational and rotational diffusion in solution

The dependence of molecular translational and rotational diffusion coefficients on the (effective) hydrodynamic radius (${R}_{\mathrm{h}}$) in solution is described by the well-known Stokes–Einstein (Einstein 1905) and the Debye–Stokes–Einstein equations (Debye 1929), respectively:

1 $${D}_{\mathrm{t}}=\frac{{k}_{\mathrm{B}}T}{6\pi \eta {R}_{\mathrm{h}}},$$

2 $${D}_{\mathrm{r}}=\frac{1}{6{\tau }_{\mathrm{c}}}=\frac{{k}_{\mathrm{B}}T}{8\pi \eta {R}_{\mathrm{h}}^{3}},$$

where *k*_B_ is the Boltzmann constant, *T* is the absolute temperature, and *η* is the viscosity of the solution. Equations 1 and 2 indicate that the translational diffusion coefficient is inversely proportional to the effective hydrodynamic radius, *R*_h_, of the molecule whereas the rotational correlation time, *τ*_c_, is proportional to ${R}_{\mathrm{h}}^{3}$, i.e. the effective volume. In other words, when translational and rotational diffusion of biomolecules are coupled, the rotational correlation time would be generally considered to be significantly more sensitive than translational diffusion coefficient in probing changes of protein size/mass as a consequence of self-association, aggregation, etc.

### Measuring molecular translational diffusion in solution by PGSE NMR

Effects of flow in NMR spectroscopy have been extensively explored with substantial advances achieved in quantitative mapping of fluid velocity with spatial resolution (Callaghan and Xia 1991; Pope and Yao 1993) and in application to angiography of blood vessels (Hartung et al. 2011). In particular, pulsed gradient spin echo (PGSE) NMR, since its introduction in the 1960s, has evolved into a key methodology for non-invasively probing molecular translational diffusion and associated properties, with applications spanning most disciplines where molecular motion is studied, including chemical engineering and biomedicine. For example, PGSE NMR-based measurements of water translational diffusion has been used to probe microstructure of porous materials (Stallmach and Karger 1999) and to serve as an imaging contrast agent, termed as diffusion-tensor MRI (Basser and Jones 2002). Both theoretical background as well as technical and practical aspects of PGSE NMR have been extensively reviewed (Price 1997, 1998), including various applications, for example in lipidic cubic phases (Lindblom and Oradd 1994; Momot and Kuchel 2003; Rajput et al. 2022). Basically, molecular self-diffusion (random walk) results in a loss of magnetization coherence in the presence of a field gradient. In the absence of flow, the application of a pair of linear pulsed gradients (d*B*_0_/d*z*) results in attenuation of the NMR signal. This signal attenuation caused by (unrestricted) molecular translational self-diffusion for a molecule with a diffusion coefficient *D*_t_ in the presence of a pair of pulsed field gradients can be expressed as

3 $$I={I}_{0}\mathrm{exp}\left\{-{\gamma }^{2}{g}^{2}{\delta }_{e}^{2}\left(\Delta -\frac{{\delta }_{e}}{3}\right){D}_{\mathrm{t}}\right\},$$

where *γ* is the spin (^1^H) gyromagnetic ratio, and *g*, *δ*_e_ and Δ are the amplitude, effective duration and separation of the gradient pulses, respectively.

### Evaluating molecular rotational reorientation via NMR relaxation measurements: ^15^N relaxation parameters of protein backbone amides

Exploring molecular motions/dynamics via NMR relaxation measurements can be traced back to the very early days when NMR was first discovered. Depending on the systems under study and the measured spin relaxation parameters, such as longitudinal or spin–lattice (*T*_1_) and transverse or spin–spin (*T*_2_) relaxation times, various factors, for instance multiple spin dipole–dipole interactions, cross-relaxation, chemical shift anisotropy, etc., will contribute to experimentally measured spin relaxation parameters. Consequently, although the theory of NMR spin relaxation is well established, a reliable molecular rotational correlation time may not be readily extracted from the measured spin relaxation parameters in the absence of exhaustive modelling. For example, abundant ^1^H networks in the surrounding vicinity would significantly complicate the ^1^H relaxation parameters. In the last 2 decades, measurements of backbone ^15^N *T*_1_, *T*_2_, and steady state {^1^H}-NOE of uniformly isotope-enriched proteins, at one or multiple fields, have emerged as a thriving methodology for exploring protein backbone dynamics with residue specificity (Kay et al. 1989; Palmer 2016). In addition to uniform ^15^N labelled material being readily available for NMR structural studies of proteins, backbone ^15^N relaxation parameters are dominated by dipole–dipole interaction to the bonded hydrogen and, therefore, can be treated as a two-spin (H–N) system, which represents one of the simplest relationships between measured spin relaxation parameters and the overall molecular rotational correlation and local dynamics. As an alternative to a direct (reduced) spectral density mapping for the evaluation of residue-specific backbone dynamics prior to (or in the absence of) a global rotational correlation time is defined (Farrow et al. 1995), a so-called model-free formalism is commonly adopted for the analysis of experimentally measured ^15^N relaxation parameters. Based on the spectral density function, i.e. the Fourier transform of the correlation function, with the approximation that the dipole–dipole interactions from nuclei other than directly bonded protons to be negligible, commonly measured backbone ^15^N relaxation rates in the laboratory frame are expressed as

4 $${R}_{1}= \frac{1}{{T}_{1}}= {d}^{2}\left[J\left({\omega }_{\mathrm{H}}-{\omega }_{\mathrm{N}}\right)+3J\left({\omega }_{\mathrm{N}}\right)+6J\left({\omega }_{\mathrm{H}}+{\omega }_{\mathrm{N}}\right)\right]+{c}^{2}J\left({\omega }_{\mathrm{N}}\right),$$

5 $${R}_{2}= \frac{1}{{T}_{2}}=\frac{{d}^{2}}{2}\left[4J\left(0\right)+J\left({\omega }_{\mathrm{H}}-{\omega }_{\mathrm{N}}\right)+3J\left({\omega }_{\mathrm{N}}\right)+6J\left({\omega }_{\mathrm{H}}\right)+6J\left({\omega }_{\mathrm{H}}+{\omega }_{\mathrm{N}}\right)\right]+ \frac{1}{6}{c}^{2}\left[3J\left({\omega }_{\mathrm{N}}\right)+4J\left(0\right)\right],$$

6 $$\mathrm{NOE}=1+\left\{\left(\frac{{\gamma }_{\mathrm{H}}}{{\gamma }_{\mathrm{N}}}\right){d}^{2}\left[6J\left({\omega }_{\mathrm{H}}+{\omega }_{\mathrm{N}}\right)-J\left({\omega }_{\mathrm{H}}-{\omega }_{\mathrm{N}}\right)\right]\right\}{T}_{1},$$

where ${d}^{2}=\frac{1}{4} {\left(\frac{{\mu }_{0}{\gamma }_{\mathrm{H}}{\gamma }_{\mathrm{N}}h}{2\pi }\right)}^{2}{\left(\frac{1}{\langle {r}_{\mathrm{NH}}^{3}\rangle }\right)}^{2}$ and ${c}^{2}=\frac{1}{3}{\omega }_{\mathrm{N}}{\Delta \sigma }^{2}$ with *μ*_0_ being the permeability of vacuum, *h* being Planck’s constant, *γ*_H_ and *γ*_N_ being the gyromagnetic ratios of ^1^H and ^15^N, respectively, *r*_NH_ = 1.02 Å being the H–N bond length, *ω*_H_ and *ω*_N_ being Larmor frequencies of ^1^H and ^15^N, respectively, and ∆*σ* = (${\sigma }_{\| }-{\sigma }_{\perp }$) =  − 160 ppm being the chemical shift anisotropy of an ^15^N nucleus. In the model-free formalism, assuming no cross-correlation between residue-specific internal motion and the global motion of the molecule, the spectral density function takes the following form (Lipari and Szabo 1982a, b):

7 $$J\left(\omega \right)=\frac{2}{5} \left[\frac{{S}^{2}{\tau }_{\mathrm{m}}}{1+{\omega }^{2}{\tau }_{\mathrm{m}}^{2}}+\frac{\left(1-{s}^{2}\right)\tau }{1+{\omega }^{2}{\tau }^{2}}\right],$$

where *S*^2^ is the order parameter and $\frac{1}{\tau }=\frac{1}{{\tau }_{\mathrm{f}}}+\frac{1}{{\tau }_{\mathrm{m}}}$ with *τ*_m_ being the global rotational correlation time of the protein and *τ*_*f*_ being the effective internal correlation time of individual backbone amides. For the analysis of ^13^C relaxation of macromolecules (polymers or lipids) based on the two-step model, the overall correlation (slow) time constant is usually estimated from relaxation parameters measured on a different nucleus, such as ^14^N, or ^2^H and ^17^O when isotope-labelled nuclei are made available (Soderman and Henriksson 2020; Wong et al. 1989). In contrast, for studies of protein backbone dynamics via ^15^N relaxation measurements, the overall correlation times of proteins, including a full rotation diffusion tensor when its 3D structure is available, are commonly estimated from a sub-group of backbone amides that do not exhibit slow internal motion and are not involved in chemical/conformational exchange process (Kay et al. 1989). An improved scheme for the estimation of isotropic correlation times, using the above-mentioned subgroups of backbone amides and including additional restraints from ^15^N NOEs, has also been described (Yao et al. 1998). While ^13^C relaxation measurements of proteins, e.g. via selectively isotope-enriched methyl groups, have seen applications in probing protein sidechain dynamics, application to the evaluation of protein overall rotational motion is complicated due to the presence of ^13^C–^13^C coupling in uniformly labelled proteins which are commonly used in NMR structural studies. Hence, protein overall rotation correlation times derived from ^15^N relaxation parameters of backbone amides often are used for the analysis of ^13^C relaxation-based studies of protein sidechain dynamics (Jin et al. 2003).

## Technical aspects and applications

### Protein self-diffusion in aqueous solution measured by PGSE NMR

#### PGSE NMR with water suppression

**Fig. 1:**
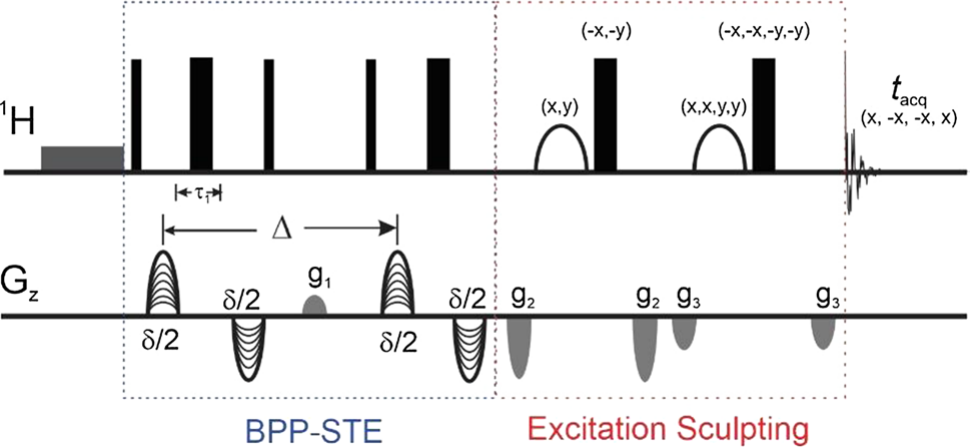
Schematic diagram of a BPP-STE PGSE NMR sequence after the incorporation of the excitation sculpting scheme for water suppression suitable for measuring protein translational diffusion coefficients in aqueous solution (Yao et al. 2018). As a convention, narrow and wide filled bars represent 90° and 180° rf-pulses, respectively. The selective 180° pulses within the excitation sculpting scheme are shown in parabolic shape. Phases of rf-pulses are *x*, unless indicated. Gradient pulses used for diffusion encoding and decoding are marked with curved lines whereas the spoiled gradient (*g*_1_) and gradients (*g*_2_ and *g*_3_) used in the excitation sculpting scheme are coloured grey.

An extensive library of PGSE NMR sequences has been developed to meet with an increasing expansion of applications. As for NMR structural studies of proteins in solution, solvent suppression is a prerequisite for measuring translational diffusion coefficients of proteins by PGSE NMR. Several robust water suppression schemes are pulsed gradient based, such as WATERGATE (Piotto et al. 1992) and excitation sculpting (Stott et al. 1995) schemes for 1D and 2D homonuclear experiments; and the echo/anti-echo scheme, originally designed for sensitivity improvement (Palmer et al. 1991), for coherence selection in heteronuclear multidimensional experiments. PGSE NMR sequences, employing WATERGATE and excitation sculpting schemes to achieve water suppression, for measuring translational diffusion coefficients of proteins in aqueous solution have been introduced (Balayssac et al. 2009; Price et al. 2002). A stimulated echo sequence, featuring bipolar pulse pair (BPP-STE) after appending an excitation sculpting segment before signal acquisition, suitable for translational diffusion measurement of proteins in aqueous solution is shown in Fig. 1. The sequence also contains a weak presaturation pulse, for further improving the efficiency of water suppression, throughout the entire range of pulsed gradients used for PGSE NMR experiments. This weak presaturation pulse is beneficial when a single axis gradient pulse, e.g. *G*_z_, is used for both diffusion encoding/decoding and water suppression, which is commonly true for spectrometers equipped with cryoprobes devoted to biological NMR. For the BPP-STE sequence shown in Fig. 1, which is less susceptible to instrumental imperfection and sample complexity in comparison to the standard STE sequence, the diffusion-induced signal attenuation in the presence of field gradient is given by

8 $$I={I}_{0}\mathrm{exp}\left\{-{\gamma }^{2}{g}^{2}{\delta }_{e}^{2}\left(\Delta -\frac{{\delta }_{e}}{3}-\frac{{\tau }_{1}}{2}\right){D}_{t}\right\},$$

where *τ*_1_ is the time interval between the bipolar gradient pulses (within the encoding or decoding, see Fig. 1). When *τ*_1_ is sufficiently shorter than *δ*_e_, Eq. 8 reduces to Eq. 3. It is worth noting that for the analysis of complex mixtures by PGSE NMR and for assessing or monitoring changes of protein translational diffusion under various conditions such as different stages of folding/unfolding, a partial region of the spectrum containing resonances arising from the protein or even a single protein resonance would be sufficient. PGSE NMR sequences involving the use of band-selective RF pulses (Yao et al. 2014a) or heteronuclear (e.g. ^13^C or ^15^N) filters for measuring translational diffusion coefficients have also been reported (Augustyniak et al. 2011; Shukla and Dorai 2011; Yao et al. 2018). One of the advantages for a selective PGSE NMR sequence is that potential dynamic range issues causing by the presence of intense resonances from molecules other than the protein of interest, such as detergents, in the solution is avoided. In addition, the presence of possible (slow) chemical exchange will complicate the interpretation of protein oligomeric state from the apparent diffusion coefficient determined by PGSE NMR (Chen et al. 1998; Johnson 1993). As a result, resonances arising from spins potentially involved in exchange, like amide protons in proteins, should be avoided for evaluating protein translational diffusion by PGSE NMR unless dedicated sequences are used. While molecular diffusion measured by PGSE NMR using nuclear spins other than protons are less likely to be susceptible to exchange, in practice they are generally less favoured because of their reduced efficacy in diffusion weighting and low signal sensitivity due to their lower gyromagnetic ratio and natural abundance. Finally, if the presence of convection in the samples is of concern, e.g. when measurements are carried out far from ambient temperatures, replacing the single STE with the double STE version can be considered so as to correct for systematic errors introduced by convection (Jerschow and Muller 1997, 1998).

#### Calibration of pulsed gradient strength and data analysis

Calibration of pulse field gradient strength is critical for comparison of experimentally determined molecular translational diffusion coefficients with those measured from other techniques, for example, dynamic light scattering. Calibrations can be carried out either on a sample containing solution with known physical dimensions or known diffusion coefficient under given conditions, such as commonly quoted diffusion coefficient of 1.90 × 10^–9^ m^2^ s^−1^ for residual H_2_O in a 100% ^2^H_2_O sample at 298.13 K (Callaghan et al. 1983; Mills 1973). The back calculation gave nearly identical calibrated values for the field gradient to those resulted from gradient profiles of known sample dimensions, e.g. internal diameter of an NMR tube (Yao et al. 2000). Extracting translational diffusion coefficients from PGSE NMR datasets involves non-linear regression analysis of signal intensities using Eq. 8 or similar, depending on the details of the sequences used, for example, Eq. 3 if a single pair of gradients was used instead. A popular alternative to the non-linear regression for the analysis of PGSE NMR data involves the use of an inverse Laplace transformation along the diffusion encoding/decoding dimension of the pseudo-2D dataset and the resultant spectrum subsequently termed as DOSY (diffusion-ordered spectroscopy) (Morris and Johnson 1992). This DOSY presentation is sometimes considered preferable in analyses of mixtures of small molecules where resonances arise from different species, which are separated based on their diffusion coefficients along the longitudinal axis of a DOSY plot. While the DOSY gives a magnificent spectroscopic view of molecular distribution based on their translational diffusion motions, it may be difficult to evaluate the outcomes of individual resonances (molecules) quantitatively.

#### Translational diffusion of Bax-∆C upon dimerization

**Fig. 2:**
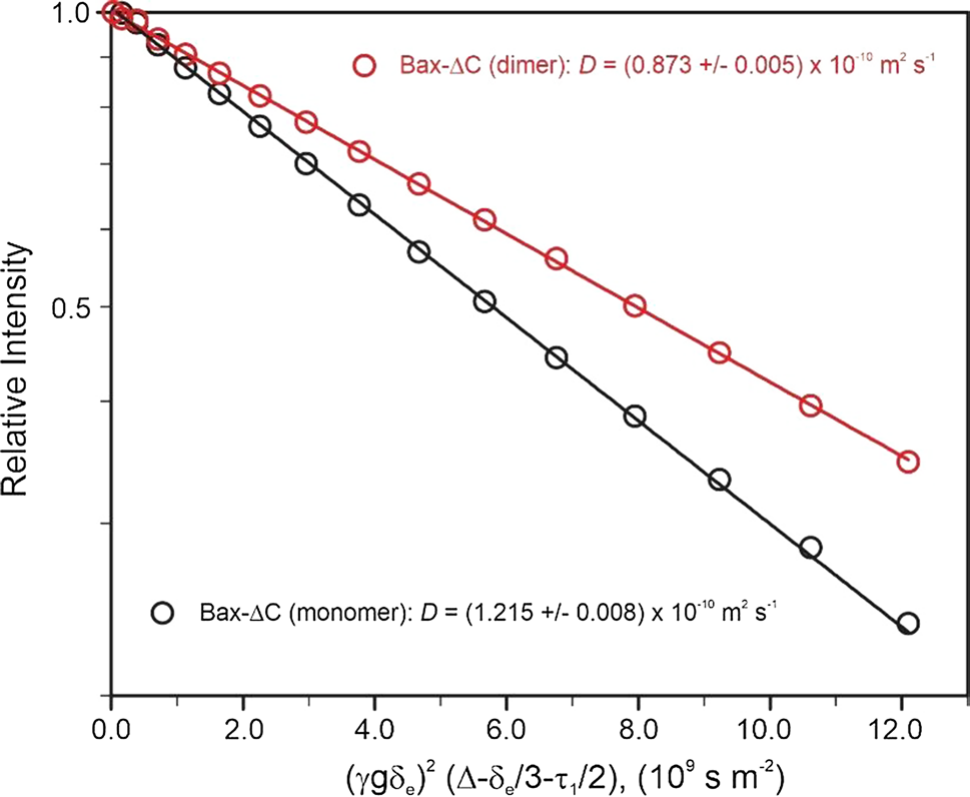
Translational diffusion coefficients of proteins in solution measured by PGSE NMR. Translational diffusion-induced signal attenuation in the presence of pulsed gradients for the monomeric and dimeric forms of Bax-∆C, a key pro-apoptotic Bcl-2 family protein. Data acquired at 305 K using a sequence similar to the one shown in Fig. 1, but with water suppression achieved using the WATERGATE scheme instead of excitation sculpting. Lines represent fits to Eq. 8. Redrawn from data in Yao et al. (2014b)

Signal attenuations in the presence of pulsed gradients for the monomeric and dimeric forms of Bax-∆C, a key pro-apoptotic Bcl-2 family protein with a construct mass of 19 kDa for its monomeric form, is shown in Fig. 2. The resultant diffusion coefficients for the monomer and dimer are (1.215 ± 0.008) and (0.873 ± 0.005) × 10^–10^ m^2^ s^−1^ at 305 K, respectively (Yao et al. 2014b). Upon dimerization, a 28% reduction of its translational diffusion motion was observed. In other words, an increase of 28% in the effective hydrodynamic radius was observed for the dimer of Bax-∆C compared to the monomer. A 26% increase in effective hydrodynamic radius is predicted by Eqs. 1 and 2 for a spherical molecule with a doubling in volume/mass. This confirmation of both monomeric and dimeric forms of Bax-∆C is in excellent agreement with results from gel filtration carried out prior to the NMR measurements (Yao et al. 2014b).

### Protein overall rotational reorientation evaluated via composite ^15^N relaxation parameters of backbone amides

#### ^15^N longitudinal (*R*_1_ = 1/*T*_1_) and transverse (*R*_2_ = 1/*T*_2_) relaxation rates

**Fig. 3:**
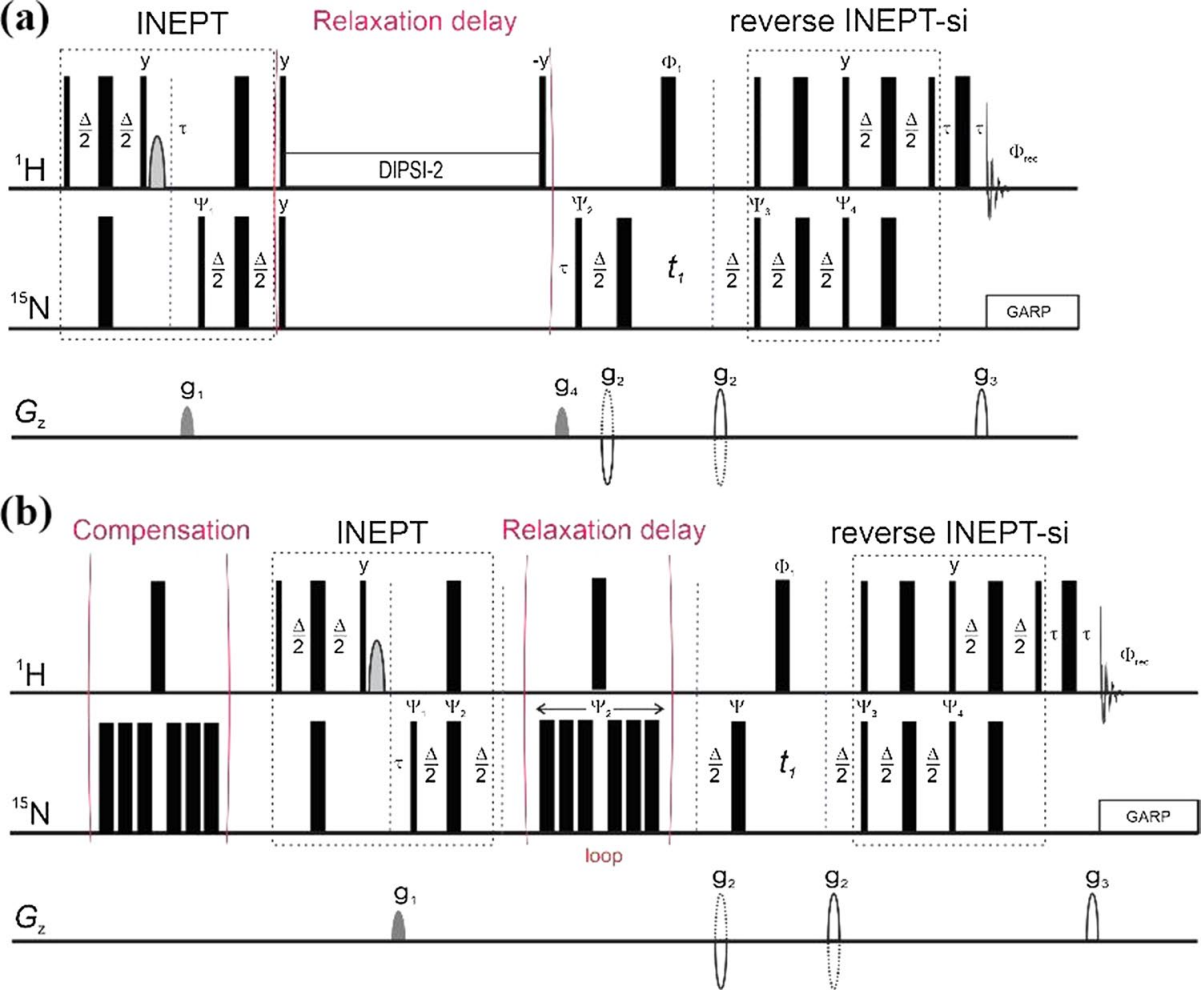
Schematic diagrams of pulse sequences for the measurement of composite backbone amide: **A** ^15^N longitudinal (*R*_1_), and **B** transverse relaxation (*R*_2_) rates. Same as Fig. 1, narrow and wide filled bars represent 90° and 180° rf-pulses, respectively. The phases for the rf-pulses are: **A**: Φ_1_ = {2(*x*),2(− *x*)}; Ψ_1_ = {4*x*,4(− *x*)}; Ψ_2_ = {*y*,− *y*}; Ψ_3_{2(*x*),2(− *x*)}; Ψ_4_ = {2(− *y*),2*y*,}; Φ_res_ = {*x*,2(− *x*),*x*,− *x*,2*x*,− *x*}, and **B** Φ_1_ = {2(*x*),2(− *x*)}; Ψ_1_ = {*x*,− *x*}; Ψ_2_ = {4(*x*),4(− *x*)}; Ψ_3_ = {2(*x*),2(− *x*)}*n*; Ψ_4_ = {2(− *y*),2*y*,}; Φ_res_ = {*x*,− *x*,− *x*,*x*}. The water flip-back rf-pulse is shown as parabolic shape filled in grey. The delay, Δ, in both the INEPT and reverse INEPT with sensitivity improvement segments are 1/(2*J*_NH_). The gradients used in echo/anti-echo coherence selection are shown in solid and dashed parabolic shape (unfilled) with the spoiler gradients filled in grey. For the measurements of collective ^15^N *R*_1_ and *R*_2_ relaxation rates, the ^15^N dimension is not acquired with the *t*_1_ delay set at ca. 6 μs.

As mentioned earlier, for the analysis of ^13^C relaxation of macromolecules, an overall correlation (slow) time of polymer/lipids is commonly estimated from relaxation parameters of a quadrupolar nuclear spin, such as ^14^N, or ^2^H and ^17^O if labelled isotopes are available in the molecules (Soderman and Henriksson 2020; Wong et al. 1989). Alternatively, ^1^H NMR relaxation times measured at low resonance frequencies have been used for studying protein tumbling as the magnetic relaxation at low frequencies is dominated by overall Brownian rotation (Krushelnitsky 2006). The presence of molecules other than the protein of interest, such as small molecules or detergents frequently present for reasons of solubility and/or stability of proteins in solution, may limit the use of these methods due to poor spectral resolution. For evaluating protein oligomeric states, as a supplement to PGSE NMR described earlier, extracting protein overall rotational correlation times from composite ^15^N relaxation parameters of backbone amides is an attractive estimation prior to a residue-specific analysis. The pulse sequences depicted in Fig. 3 involve the use of echo/anti-echo for coherence selection (similar to standard *hsqct1etf3gpsi* and *hsqct2etf3gpsi* in Bruker pulse sequence library) and are suitable for measuring composite ^15^N relaxation rates, *R*_1_ and *R*_2_, of protein backbone amides. Except for an additional heat compensation segment at the beginning in the ^15^N *T*_2_ sequence, both sequences are composed of three basic segments: (1) an initial INEPT (insensitive nuclei enhanced by polarization transfer) to transfer magnetization from ^1^H to ^15^N, (2) a variable ^15^N relaxation delay, and (3) a reverse INEPT (with sensitivity improvement) to transfer magnetization from ^15^N back to ^1^H for detection.

Composite ^15^N *R*_1_ and *R*_2_ values can be obtained by fitting the integrals of peaks across the entire amide region (typically, 7.0–10.0 ppm) to a two-parameter single-component exponential decay and a four-parameter dual-component exponential decay, respectively:

9a $$I\left(t\right)=I\left(0\right)\mathrm{exp}\left(-{R}_{1}t\right),$$

9b $$I\left(t\right)={I}^{\mathrm{f}}\left(0\right)\mathrm{exp}\left(-{R}_{2}^{\mathrm{f}}t\right)+{I}^{s}\left(0\right)\mathrm{exp}\left(-{R}_{2}^{\mathrm{s}}t\right),$$

where ${R}_{2}^{\mathrm{f}}$ and ${R}_{2}^{\mathrm{s}}$ represent the shorter and longer components of composite ^15^N transverse relaxation times, *T*_2_ = 1/*R*_2_, respectively. For a globular protein, ${R}_{2}^{\mathrm{f}}$ represents the collective contribution of backbone amides subjected to relatively restrained internal motion, such as those in and around its hydrophobic core, with *I*^f^(0) >  > *I*^s^ (0). For the estimation of protein rotational correlation time of globular proteins, ${R}_{2}^{\mathrm{f}}$ is then used as an approximation of composite ^15^N translation relaxation rate of backbone amides not experiencing significant internal motion, and the spectral density function shown in Eq. 7 reduces to $J\left(\omega \right)=\left(\frac{2}{5}\right)\frac{ {\tau }_{\mathrm{c}}}{1+{\left(\omega {\tau }_{\mathrm{c}}\right)}^{2}} .$ An estimation of rotational correlation time of a globular protein may be obtained through the ratio of collective backbone ^15^N ${R}_{2}^{\mathrm{f}}$ and *R*_1_ relaxation rates similar to that used in ^15^N relaxation-based protein backbone dynamics studies:

10 $${\tau }_{\mathrm{c}}\propto \frac{{R}_{2}^{\mathrm{f}}}{{R}_{1}}=\frac{\left(\frac{{d}^{2}}{8}\right)\left[4J\left(0\right)+J\left({\omega }_{\mathrm{H}}-{\omega }_{\mathrm{N}}\right)+3J\left({\omega }_{\mathrm{N}}\right)+6J\left({\omega }_{\mathrm{H}}\right)+6J\left({\omega }_{\mathrm{H}}+{\omega }_{\mathrm{N}}\right)\right]+\left(\frac{{c}^{2}}{6}\right)\left[4J\left(0\right)+3J\left({\omega }_{\mathrm{N}}\right)\right]}{\left(\frac{{d}^{2}}{4}\right)\left[J\left({\omega }_{\mathrm{H}}-{\omega }_{\mathrm{N}}\right)+3J\left({\omega }_{\mathrm{N}}\right)+6J\left({\omega }_{\mathrm{H}}+{\omega }_{\mathrm{N}}\right)\right]+{c}^{2 }J\left({\omega }_{\mathrm{N}}\right)}.$$

Effective rotational correlation times can then be estimated from the collective ^15^N ${R}_{2}^{\mathrm{f}}$/*R*_1_ ratios using programs, such as Modelfree (AG Palmer III, Columbia University), TENSOR2 (Dosset et al. 2000), or NMRbox (Maciejewski et al. 2017), that are publicly available for the analysis of protein dynamics based on experimentally measured NMR relaxation parameters.

#### Rotational reorientation of Bax-ΔC upon dimerization

**Fig. 4:**
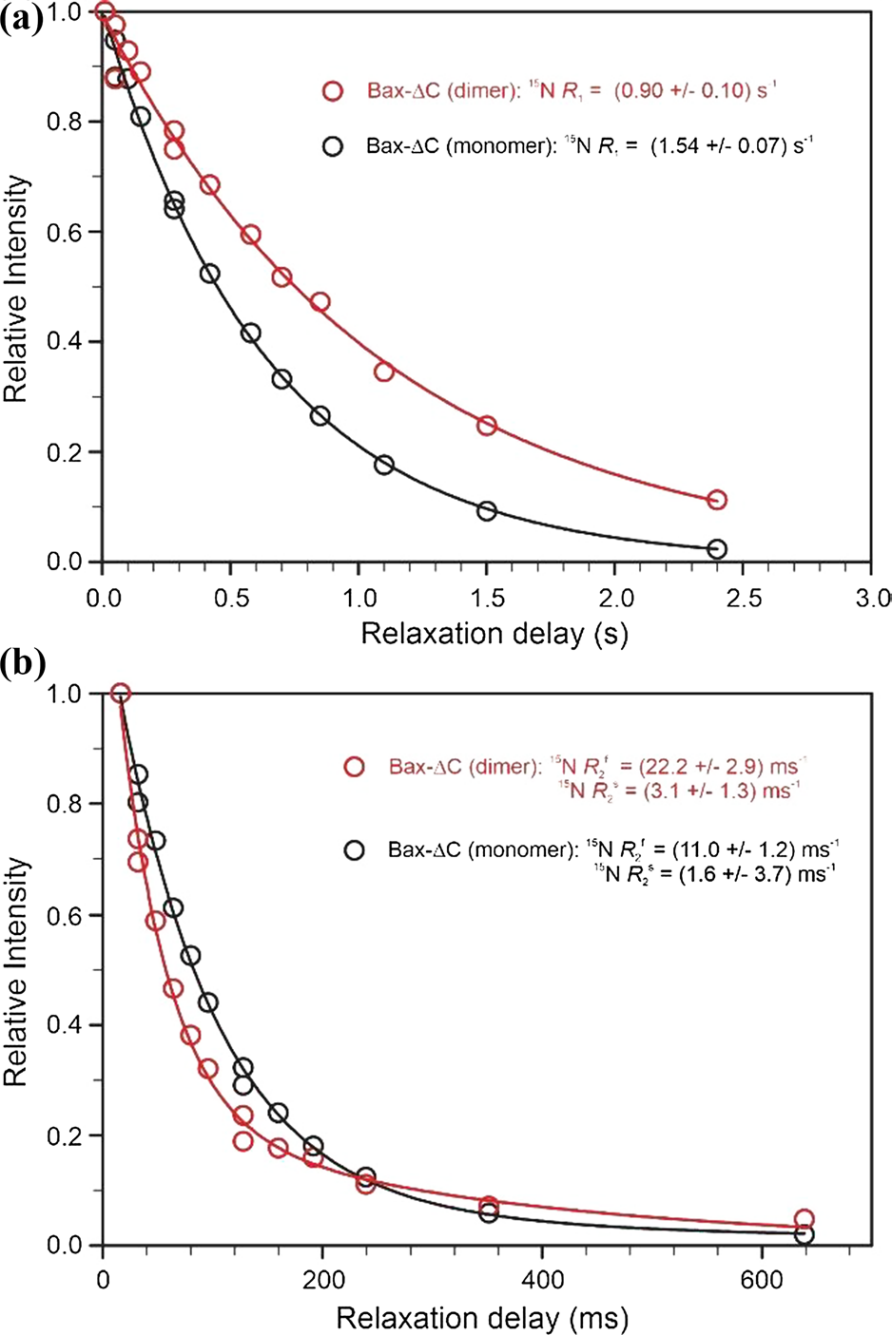
Collective backbone ^15^N relaxation parameters, *R*_1_ (**A**), and ${R}_{2}^{\mathrm{f}}$ (**B**) for monomeric (in black) and dimeric (in red) forms of Bax-ΔC. Lines represent fit to Eqs. 9a and 9b and the resultant fitted ^15^N *R*_1_ and ${R}_{2}^{\mathrm{f}}$ are (1.54 ± 0.07) s^−1^ and (11.0 ± 1.2) s^−1^ for the monomer and (0.90 ± 0.10) s^−1^ and (22.2 ± 2.9) s^−1^ for the dimer, respectively. Redrawn from data in Yao et al. (2014b)

An example of collective backbone ^15^N relaxation rates of monomeric and dimeric forms of Bax-ΔC protein are shown in Fig. 4. The resultant correlation for the monomeric and dimeric forms of Bax-ΔC are 9.46 and 18.68 ns, respectively, representing an increase of ca. 97% upon dimerization, which corresponds to an increase of 25.5% in effective hydrodynamics radius, *R*_h_, based on Eq. 2 and is in excellent agreement with results from PGSE NMR described earlier.

Since the composite ^15^N relaxation parameters of backbone amides are used in evaluating protein rotational correlation times, the inclusion of backbone amides experiencing local/regional flexibility or conformational exchange will impact the estimated rotational correlation times to a certain extent. If potential exchange of backbone amides is suspected, then relaxation dispersion (Palmer 2014) or chemical shift saturation transfer (Vallurupalli et al. 2017) experiments could be used to identify those backbone amides and subsequently exclude them from the composite evaluation. Second, an accurate description of rotational diffusion anisotropy is critical for subsequent analysis of protein backbone (typically by ^15^N relaxation) and sidechain dynamics (by ^13^C relaxation). The difference in terms of overall rotation correlation time, however, is often minor. In the case of murine interleukin-3, a full anisotropic rotational diffusion tensor resulted in *D*_*xx*_:*D*_*yy*_:*D*_*zz*_ of 0.51:0.89:1.0 corresponding to an effective rotational correlation time of 10.77 ns ($\tau =\frac{1}{6{D}_{r}}=\frac{1}{2\left({D}_{xx}+{D}_{yy}+Dzz\right)} ),$ compared to a value of 11.05 ns from an isotropic analysis (Yao et al. 2011).

#### ^15^N cross-correlated transverse relaxation rates of ^15^ N alpha (*R*_α_) and beta (*R*_β_) spin states of backbone amides

**Fig. 5:**
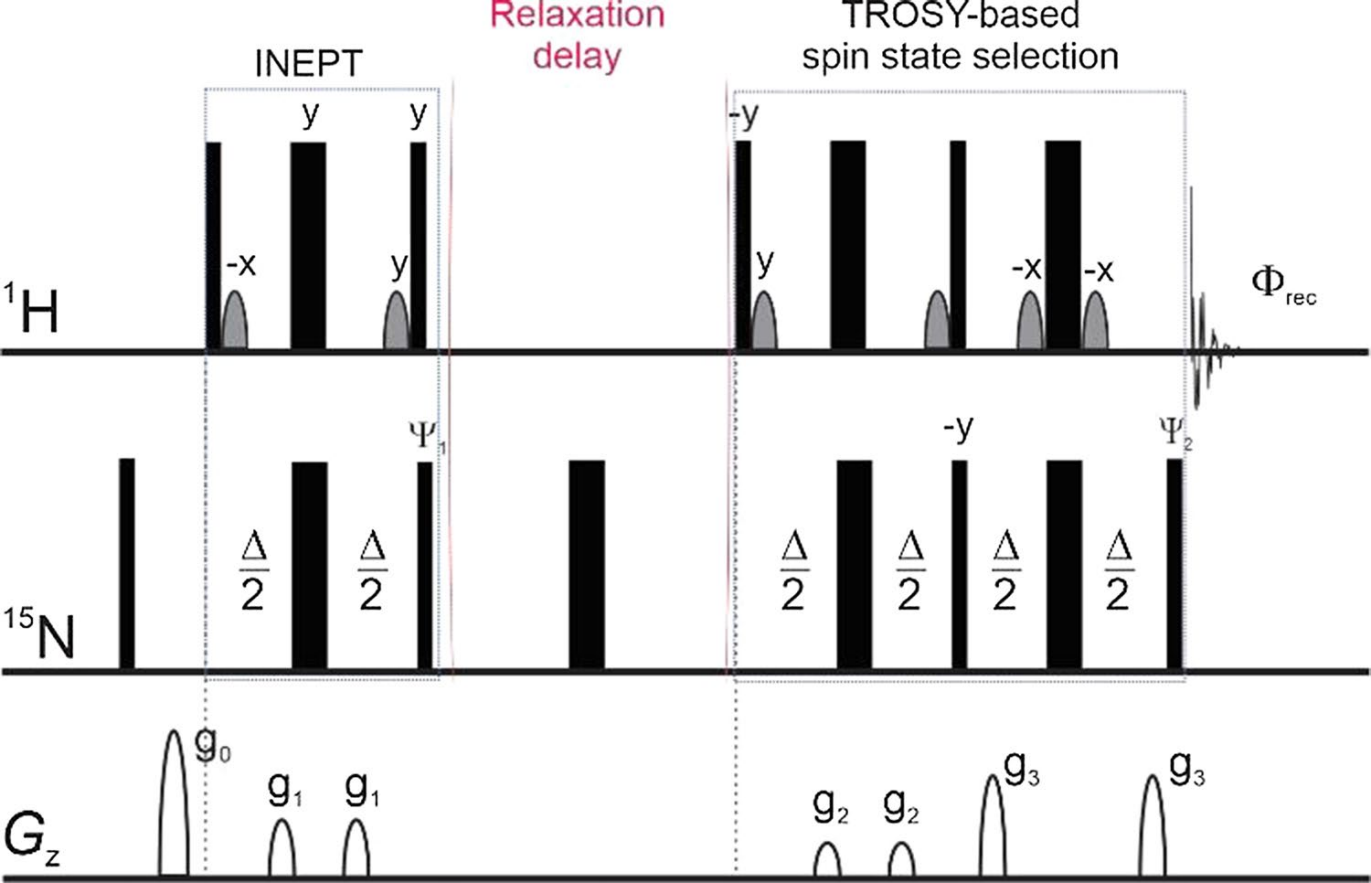
Schematic [^15^N, ^1^H]-TRACT sequence for measuring cross-correlated transverse relaxation rates of α- and β-spin state for ^15^N uniformed labelled proteins as proposed by Lee et al. (2006) As in Fig. 1, narrow and wide filled bars represent 90° and 180° rf-pulses. The selection of cross-correlated spin states is achieved via alternating phases of Ψ_1_ and Ψ_2_ as follows: Ψ_1_ = {*y*,− *y*,− *x*,*x*}, Ψ_2_ = {− *x*} for α-spin state and Ψ_2_ = {*x*} for β-spin state, respectively, and Φ_rec_ = {*y*,− *y*, *x*, − *x*}. The delay, Δ, in both the INEPT and TROSY segments are 1/(2*J*_NH_).

In the case above, composite ^15^N backbone *R*_1_ and *R*_2_ of monomeric and dimeric forms of Bax-ΔC resulted in very satisfactory agreement and confirmed by gel filtration profile used in the sample preparations. Clearly, the validity of evaluating protein rotational correlation times via composite backbone ^15^N relaxation parameters strongly depends on the compactness of the proteins under study. In other words, the significance of local motions, particularly those on slow timescale, such as conformational exchange to be specific, will impact the outcome. Furthermore, the presence of conformational exchange and the contribution of remote dipole–dipole interactions, in particular as the protein/protein complex mass increases, may significantly impact ^15^N transverse and longitudinal relaxation times. Consequently, the estimation of protein rotational correlation times via the ratio of ${R}_{2}^{\mathrm{f}}$/*R*_1_, as described above, may become untenable. A dedicated pulse sequence for measuring protein rotational correlation times via collective cross-correlated ^15^N relaxation rates, named TRACT (TROSY for rotational correlation times), has been described (Lee et al. 2006). The TRACT experiment measures ^15^N transverse cross-correlated relaxation rates of the *α*- and *β*-spin state, respectively:

11a $${R}_{\alpha }=\lambda -{\eta }_{xy}+{R}_{\mathrm{DD}}+{R}_{\mathrm{CS}},$$

11b $${R}_{\beta }=\lambda -{\eta }_{xy}+{R}_{\mathrm{DD}}+{R}_{\mathrm{CS}},$$

where *λ*, *η*_*xy*_, *R*_*DD*_, and *R*_*CS*_ are the auto-relaxation rate, the transverse cross-correlated relation rate, transverse relaxation due to dipole–dipole coupling with remote protons, and relaxation contributed from chemical exchange, respectively. The correlation time, *τ*_c_ is then calculated from the difference of ^15^N relaxation rates of the α- and β-spin states:

12 $$\frac{\left({R}_{\beta }-{R}_{\alpha }\right)}{2}={h}_{xy}=d c \left(4J\left(0\right)+3J\left({\omega }_{\mathrm{N}}\right)\right)\left(3{\mathrm{cos}}^{2}\theta -1\right),$$

where *d* and *c* are the same as defined in Eqs. 4–6 and the spectral density function takes the reduced form of Eq. 7, $J\left(\omega \right)=\left(\frac{2}{5}\right)\frac{ {\tau }_{\mathrm{c}}}{1+{\left(\omega {\tau }_{\mathrm{c}}\right)}^{2}}$, as described earlier. Angle *θ* is defined by the N–H bond and the unique axis of ^15^N chemical shift tensor, which is assumed to be axially symmetric. Clearly, the contribution of dipole–dipole coupling from remote protons and chemical exchange are eliminated in Eq. 12, which make the TRACT sequence superior to the basic ^15^N *T*_1_- and *T*_2_-based sequences as conventionally used for probing protein backbone dynamics via ^15^N relaxation parameters in the laboratory frame. A schematic diagram of the TRACT sequence, which adopts a TROSY scheme in the selection of spin state for detection, instead of a reverse INEPT as seen in conventional ^1^H–^15^N HSQC (Fig. 3), is shown in Fig. 5 (Lee et al. 2006).

#### Rotational correlation time of the elonginBC and the SOCS box domain complex

**Fig. 6:**
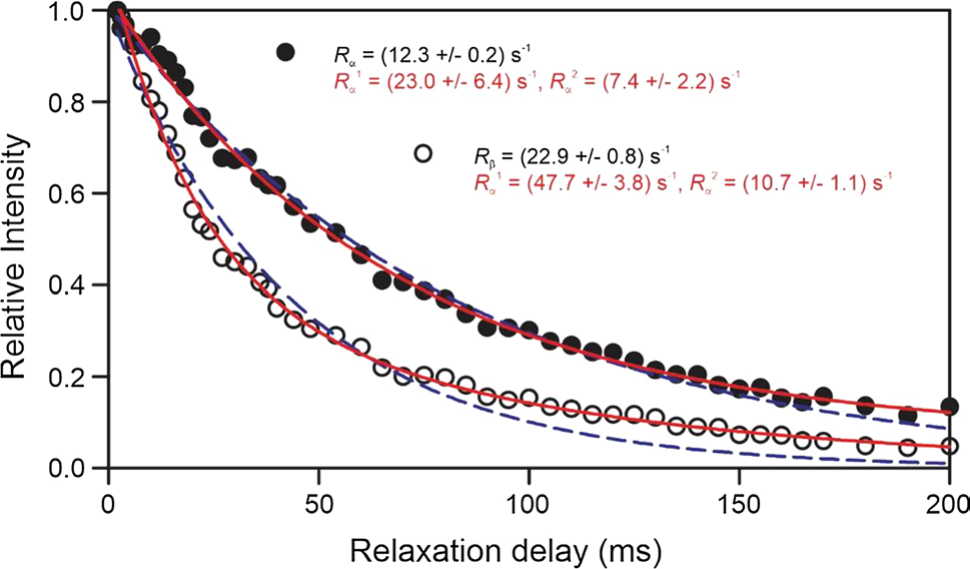
Composite ^15^N cross-correlated transverse relaxation rates of α- and β-spin states for elonginBC in complex with the SOCS box domain of SOCS3 measured at 293 K for estimation of its rotation correlation time. Lines represent fits to single exponential (in blue) and dual-exponential decay (in red). Redrawn from data in Yao et al. (2008)

Unlike the Bax-ΔC system shown earlier, for a 28 kDa complex formed by elonginBC and the SOCS box domain of SOCS3 (Babon et al. 2008), the laboratory frame composite backbone ^15^N *R*_1_ and *R*_2_ failed to produce a sufficiently reliable *R*_2_ value for backbone amides, presumably due to substantial internal motion. The cross-correlated transverse relaxation rates of α- and β-spin states, *R*_*α*_ and *R*_*β*_, as measured using the TRACT sequences, resulted in a lower limit value for *τ*_c_ of 15.4 ns at 293 K (Yao et al. 2008). Collective backbone ^15^N cross-correlated transverse relaxation rates of the elonginBC-SOCS box domain complex acquired using the TRACT sequence (Fig. 5) are shown in Fig. 6. The slight deviation from single exponential decay in both cross-correlated transverse relaxation rates are evident in Fig. 6 and reflect contributions to the composite profiles of ^15^N cross-correlated transverse relaxation rates from specific backbone amides undergoing relatively slower motions. It has been suggested that a narrow spectral region within the amide proton chemical shift range, that contains only dispersed resonances arising from the folded fraction of protein, might minimize the interference (Fuglestad et al. 2017). The TRACT sequence has also been employed to evaluate proteins in a DHPC micelle complex (Edrington et al. 2011) and reverse micelles (Nucci et al. 2011). Recently, a new algebraic solution for determining overall rotational correlation times from cross-correlated transverse relaxation rates of spin-state α and β as measured using the TRACT sequence has been reported (Robson et al. 2021).

### Internal standard for diffusion coefficient and hydrodynamic radius

In theory, explicit molecular mass may be calculated from experimentally obtained translational diffusion coefficients when detailed solution properties, such as viscosity, and the shape factor of the proteins together with the amount of bound water, etc. are known (Cantor and Schimmel 1980). Assessing protein oligomeric state usually involves comparing translational diffusion coefficients measured over time and/or over different solution conditions. To avoid complications arising from changes of solution conditions, small molecules, either already existing in the solution or purposely introduced, have been used as internal viscosity standards for PGSE NMR measurements (Chen et al. 1995; Jones et al. 1997). These internal viscosity standards may also serve as a hydrodynamic radius reference if known for the reference molecule, e.g. dioxane with a calculated *R*_h_^Ref^ of 2.12 Å, is frequently quoted (Wilkins et al. 1999). The apparent hydrodynamic radius of the protein, *R*_h_^pro^ can then be calculated based on the Stokes–Einstein equation (Eq. 1):

13 $${R}_{\mathrm{h}}^{\mathrm{pro}}={R}_{\mathrm{h}}^{\mathrm{Ref}}\left(\frac{{D}_{t}^{\mathrm{Ref}}}{{D}_{t}^{\mathrm{pro}}}\right).$$

This approach was employed in comparing native and urea-denatured lysozyme by PGSE NMR diffusion measurements to avoid the complexities arising from variations in solution concentration and temperature (Jones et al. 1997). For example, the explicit effective hydrodynamic radii of murine interleukin-3 in two buffer solutions over a temperature range from 283 to 303 K were obtained by measuring translational diffusion coefficients of both the reference molecule, dioxane, and murine interleukin-3 (Yao et al. 2011). The dynamic range of diffusion-induced signal attenuation between protein and internal reference of small molecule should be considered. In other words, separate PGSE NMR experiments might have to be performed for the measurement of the protein and the internal references. Recently, a selective PGSE NMR sequence capable of encoding different species independently in a single experiment has been described (MacKinnon et al. 2021). The inclusion of small molecules as internal hydrodynamic radius references could also serve as an internal standard for evaluating rotational correlation of proteins via composite ^15^N relaxation measurements as described above. The small molecule provides a means to assess if the change of observed correlation times is not due to a variation in solution conditions, such as viscosity and pH. Small molecules other than dioxane may also be used as an internal reference as long as they do not interact with the protein or other solutes molecules in the solution. While dioxane has been frequently used as an internal reference of hydrodynamic radius in aqueous solution (Wilkins et al. 1999), for high protein concentrations, it may be not a suitable probe for viscosity experienced by the protein (Rothe et al. 2016).

## Conclusion

As a spectroscopic method, NMR provides a vital means for evaluating both translational and rotational motions of molecules in solution, a predominant mode for numerous characterizations of biomolecules. We have presented a mini-review on the evaluation of biomolecular oligomeric state in solution using NMR spectroscopy with a focus on practical aspects of PGSE NMR and composite backbone ^15^N relaxation-based methods. Although protein translational and rotational diffusion may be decoupled under certain conditions, such as lateral diffusion in membranes (Macdonald et al. 2013) or under molecular crowding (Roos et al. 2015), in this review, translational and rotational correlation are considered as remaining coupled, i.e. both Eqs. 1 and 2 hold true, as is the case for the majority of protein structural, dynamic, binding and functional studies in aqueous solution by NMR. While the molecular rotational correlation time is more sensitive than translational motion to changes in biomolecular oligomeric state, due to its indirect experimental accessibility, the rotational correlation time is more susceptible to experimental uncertainties or error. In contrast, direct measurement of molecular translational diffusion by PGSE NMR is readily applied. Nevertheless, both methods provide supplementary experimental means for assessing protein oligomeric state in solution. However, we emphasize that changes in experimentally determined values of *D*_t_ and *τ*_c_ can occur in the absence of protein oligomerization, such as partial or extensive unfolding. Therefore, caution is needed in the interpretation of both translational and rotational parameters of proteins measured by NMR: the effects of anisotropy may be quite reduced for the overall apparent *τ*_c_, but be more substantial for *D*_t_. Other NMR spectral parameters should also be considered when changes are observed in *D*_t_ and *τ*_c_. For example, partial unfolding of proteins is generally accompanied by significant difference in spectral features compared to structured proteins. Finally, when high-resolution 3D protein structures are available, tools for structure-based hydrodynamic calculations of protein translational and rotational motion can be applied (de la Torre et al. 2000; Rezaei-Ghaleh et al. 2013). PGSE and composite ^15^N relaxation NMR measures, however, provide an additional quality check for ensuring experimental conditions of protein binding, interaction, screening, etc., are biologically relevant. An improved quantitation of protein rotational diffusion may become accessible by a full anisotropy analysis of rotational motion (Palmer 2004) or an alternative approach for the determination of rotational correlation time can be explored if necessary (Korchuganov et al. 2004).

## Abbreviations

BPP-STE: Bipolar pulse pair stimulated echo
INEPT: Insensitive nuclei enhanced by polarization transfer
MRI: Magnetic resonance imaging
NMR: Nuclear magnetic resonance
NOE: Nuclear Overhauser effect
PGSE: Pulsed gradient spin echo
STE: Stimulated echo
TRACT: TROSY for rotational correlation times
TROSY: Transverse relaxation optimized spectroscopy
